# Flavonoids and Wnt/β-Catenin Signaling: Potential Role in Colorectal Cancer Therapies

**DOI:** 10.3390/ijms150712094

**Published:** 2014-07-08

**Authors:** Nathália G. Amado, Danilo Predes, Marcela M. Moreno, Igor O. Carvalho, Fábio A. Mendes, José G. Abreu

**Affiliations:** Biomedical Science Institute, Federal University of Rio de Janeiro, Bloco F2 Sala 15, Rio de Janeiro 21949-590, Brazil; E-Mails: nathalia@icb.ufrj.br (N.G.A.); danilopredes@gmail.com (D.P.); celammoreno@gmail.com (M.M.M.); igor.oliveira93@outlook.com (I.O.C.); mendes@icb.ufrj.br (F.A.M.)

**Keywords:** β-catenin, quercetin, colorectal cancer, isoquercitrin, polyphenols, small molecules

## Abstract

It is now well documented that natural products have played an important role in anticancer therapy. Many studies focus on the ability of these natural compounds to modulate tumor-related signaling pathways and the relationship of these properties to an anticancer effect. According to the World Health Organization (WHO), colorectal cancer (CRC) is the third most common cancer and the fourth leading cause of cancer death among men and women. Therefore, finding strategies to fight against CRC is an emergent health problem. CRC has a strong association with deregulation of Wnt/β-catenin signaling pathway. As some types of natural compounds are capable of modulating the Wnt/β-catenin signaling, one important question is whether they could counteract CRC. In this review, we discuss the role of flavonoids, a class of natural compounds, on Wnt/β-catenin regulation and its possible potential for therapeutic usage on colorectal cancer.

## 1. Introduction

The theory that natural molecules might inhibit cancer development started more than 100 years ago, when laboratories began to investigate the effect of small molecules extracted from plants and fungi on tumor development and growth [1,2,3]. Currently, many studies are focusing on the ability of these natural compounds to modulate signaling pathways and to control fundamental cellular functions such as cell proliferation, cell differentiation and cell death [4,5,6]. These studies have improved the current knowledge about the molecular mechanism that regulate the action of compounds from natural extract, and provided better understanding of signaling pathways functioning in healthy and pathological conditions [5,6,7,8].

Uncontrolled cell growth has been a feature of many types of tumors, in particular colorectal cancer which is, according to the WHO, the third most common cancer with 1,361,000 cases in 2012 [9]. Despite the significant advances in discovering the details of the molecular mechanisms, current treatments consist mostly of surgery and conventional chemotherapy, which confer limited benefit [10,11]. Notably, It has been reported that a significant proportion of CRC patients use natural extracts as complementary therapies, and many of the commonly used antitumor drugs are derived from natural compounds, which are either directly extracted from plants, from other natural sources or chemically derived from natural compounds [11,12,13,14].

A great effort has been made to understand the molecular mechanism by which these natural compounds are acting to inhibit tumor growth [4,6,10,15]. Extensive research during the last half century has identified various molecular targets that can potentially be used for treatment [16]. Part of this effort has been dedicated to identifying substances from natural sources that are capable of modulating the Wnt/β-catenin signaling pathway, which plays pivotal role in CRC development, growth and metastasis [17]. In this review, we discuss the usage of chemical substances, focusing on flavonoids as Wnt/β-catenin inhibitors and their potential for therapeutic usage on colorectal cancer.

## 2. WNT/β-Catenin Signaling and Colorectal Cancer

Wnts are an evolutionary conserved family of secreted glycoproteins found as 19 distinct Wnt ligands in mammalians [18]. The Wnt/β-catenin pathway is primarily divided into two main categories based on their role in cytosolic β-catenin stabilization and upon activation of specific receptors: canonic and non-canonic Wnt signaling [19,20]. For the purpose of this review we will only focus on the canonical Wnt signaling.

**Figure 1:**
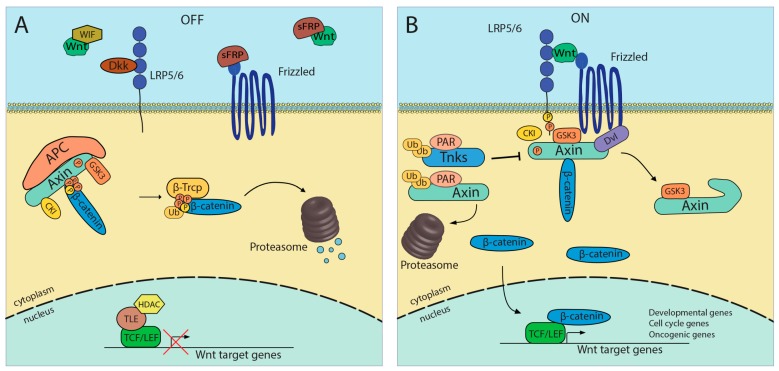
Wnt/β-catenin signaling pathway. (**A**) In absense of Wnt; (**B**) after binding of Wnt-ligand. APC, adenomatous poyposis coli; CK1, casein kinase1; Dkk, dickkopf; Dvl, disheveled; β-Trcp, beta-transducin repeat containing; GSK3, glycogen synthase kinase 3; LRP 5/6, LDL receptor-related protein 5/6; TCF, T cell factor. From APC: FRP, frizzled related protein; WIF, Wnt inhibitor factor; HDAC, histone deacetylase; Tnks, tankyrases; LEF, lymphoid enhancer-binding fator; PAR, poly (ADP-ribose); TLE, transducin-like enhancer proteins.

During canonical Wnt signaling, binding of Wnt ligands to Frizzled/low-density lipoprotein-related protein 5/6 (LRP5/6) receptor complexes causes stabilization of β-catenin. Activation of the Wnt pathway is controlled by regulation in disassembly of the destruction complex, which stabilizes β-catenin. The destruction complex, composed of APC (Adenomatous polyposis coli), Axin1, GSK-3β (Glycogen synthase kinase 3-β), and CK1 (casein kinase 1), phosphorylates serine residues in β-catenin leading to its ubiquitination by β-Trcp (F-box/WD repeat-containing protein 1A) and degradation by the proteasome. Stabilized β-catenin is then able to translocate to the nucleus and, through interactions with the T-cell factor (Tcf)/lymphoid enhancer factor 1 (LEF-1), modulates the expression of specific target genes that control developmental and cell cycle genes and oncogenes (Figure 1) [20,21]. Thus, the importance of this pathway is revealed by its crucial role in embryonic axis establishment, cell fate determination, maintenance of adult tissue homeostasis and regeneration [21,22,23]. It is noteworthy the multiplicity of fine regulation mechanisms that Wnt/β-catenin pathway requires in physiological conditions [22].

Conversely, mutations in components of the Wnt/β-catenin are often associated with cancer, in particular in CRC [24,25]. The mutations can be inherited or are acquired, and most likely occur in the intestinal crypt stem cells [25,26]. The most common alteration of the pathway includes mutation on APC, β-catenin and TCF/LEF [27,28,29,30,31,32,33]. Additional mechanisms that lead to uncontrolled regulation of Wnt signaling and that contribute to tumor progression remain unknown.

**Figure 2:**
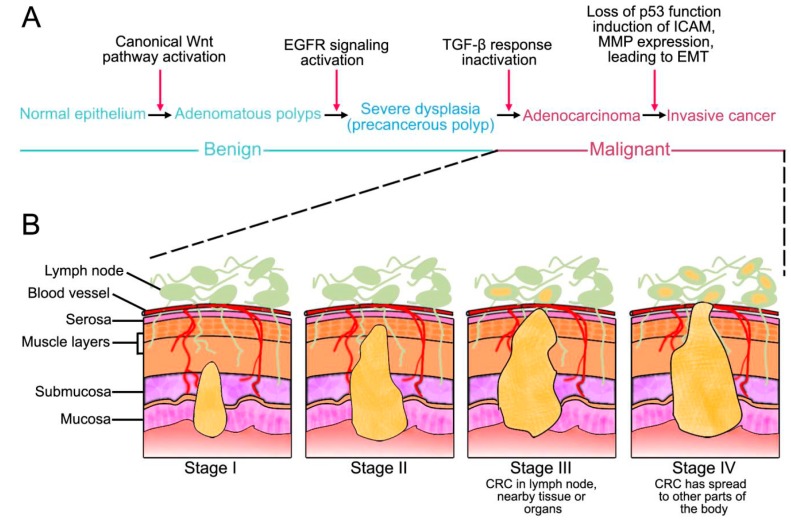
Scheme of colon cancer cells progression. (**A**) The progression of mutations that commonly lead to colorectal cancer; (**B**) Scheme that represent the four stage of colorectal cancer (CRC): Stage I–IV: Note that CRC in Stage IV is in lymph node and has spread to at least one distant organ.

CRC is associated with polyps, not detected in early stages, leading to tumor development [34]. Polyps have different structures and distributions along the intestinal mucosa and histological analysis indicates their potential to develop cancer. CRC development and progression involve silencing of genes and alteration of cellular and molecular mechanisms that originally inhibit tumorigenesis. According to World Health Organization (WHO), colorectal cancer progression is classified into five stages related with morphological and molecular changes. The stages are expressed in Roman numerals from stage 0 (the least advanced) to stage IV (the most advanced) (National Cancer Intitute) [35,36] (Figure 2).

In the stage 0, cancer is in the earliest stage and it has not grown beyond the inner layer (mucosa) of the colon or rectum. This stage is also known as carcinoma *in situ* or intramucosal carcinoma [30,35]. In stage I, cancer has grown through the muscularis mucosae into the submucosa or it may also have grown into the muscularis propria. It has not spread to nearby lymph nodes or distant sites [35] (Figure 2). During stage II, the tumor grows into the outermost layers of the colon or rectum. However, it has not reached nearby organs and has not yet spread to the nearby lymph nodes or distant sites [31,35]. When stage III is achieved, cancer grows through the mucosa into the submucosa and it may also have reached into the muscularis propria. The tumor has spread to at least one nearby lymph node or to areas of fat near the lymph nodes [35] (Figure 2). In the most advanced, stage IV, cancer may or may not grow through the wall of the colon or rectum, but always spreads to either lymph nodes, distant parts of the peritoneum (the lining of the abdominal cavity) or distant organs (such as the liver or lung) (Figure 2).

A recent analysis published in 2012 by The Cancer Genome Atlas Network, has uncovered several critical genes and important pathways in the initiation and progression of CRC [35]. These include the *Wnt*, *RAS2MAPK*, *PI3K*, *TGF-β*, *p53* and DNA mismatch-repair pathways [35] They described that over 94% of CRC had a mutation in one or more members of the Wnt/β-catenin signaling pathway, predominantly in the *APC* gene. Without *APC*, β-catenin accumulates to high levels, translocates into the nucleus, associates with TCF/LEF leading to its binding to DNA and consequent transcription of genes that are important for stem cell renewal and differentiation. However, when inappropriately expressed, APC can lead to cancer [37]. Another 5% of colorectal cancers have activating mutations in the β-catenin [37]. In addition, various sets of analyzed tumors showed changes in TCF/LEF-encoding genes, suggesting additional role for TCF/LEF transcription factors in CRC beyond being partners of β-catenin [35,37]. The relationship between Wnt/β-catenin and CRC suggests that Wnt inhibitors can be used as potential therapeutic approaches against CRC. Therefore, we highlight the effect of synthetically designed or natural extracted small molecules on Wnt/β-catenin signaling pathway modulation. In the next sections, we discuss the role of small molecules on Wnt signaling and the potential effects of these compounds against colorectal cancer growth.

## 3. Small Molecules as Wnt Inhibitors: Protein–Drug Interaction

Since Wnt signaling is critical for some carcinogenic development, many studies have been searching molecules capable of modulating this signaling pathway [38]. Several new compounds have recently been described as inhibitors of different components of the Wnt/β-catenin signaling pathway (Table 1). Pyrvinium, an anthelmintic drug, has been shown to interact with and activate CK1β, which will form a complex with APC, Axin and GSK3β, leading to β-catenin degradation and inhibition of Wnt signaling. The role of Pyrvinium as Wnt signaling inhibitor coincides with the inhibition of cell proliferation in HCT116 and SW480 cell lines [39]. Other small molecules, such as Olaparib (AZD2281), XAV939, JW55 and JW74, block Wnt/β-catenin signaling in colon cancer cell lines by binding to tankyrase (TNKS) catalytic poly-ADP-ribose polymerase (PARP) domain, which results in dramatic stabilization of the Axin protein, thereby lead to increased β-catenin destruction [40]. The Wnt signaling inhibition by JW55 reduces tumor growth *in vitro* and *in vivo* on *APC* mutant mice [41]. IWR-1 is another inhibitor of TNKS1/2 with no effect on PARP1/2 activity [40]. G007-LK and G244-LM are more examples of TNKS inhibitors [42]. Interestingly, G007-LK has great stability and sufficient bioavailability to inhibit Wnt signaling and tumor growth in xenograft tumors. Studies attempting to elucidate the essential structures of TNKS inhibitors enabled the modification and improvement of the inhibitory capacity of these molecules [43,44].

Emami KH *et al.* showed ICG-001 as a selective inhibitor of Wnt/β-catenin signaling through specific binding of CREB-binding protein (CBP), disrupting its interaction with β-catenin leading to Wnt signaling inhibition [45]. While windorphen (WD) has been described as a Wnt inhibitor that specifically targets p300 histone acetyltransferase, disrupting its association with β-catenin [46]. NSC668036, 3289-8625, FJ9, Pen-N3 inhibit Dishevelled (Dsh) specifically binding to its PDZ domain [17,47,48,49]. This binding disrupts Dsh interaction with Fzd7.

Nuclear inhibitors of Wnt signaling are also very pursued, since upstream inhibition of Wnt signaling may have collateral effects on others signaling pathways. iCRT3, iCRT5, iCRT14, PKF118-310, PKF115-584 and CGP049090, BC21 are capable of inhibiting Wnt signaling by disrupting the interaction of β-catenin with its transcription factor TCF4 [10,50,51]. iCRT14 was also capable of interfering on TCF binding to DNA [51]. NC043 may also, directly or indirectly, disrupt β-catenin/TCF4 association [52]. CCT031374, CCT036477, and CCT070535 inhibit Wnt signaling at the β-catenin level, but independent of β-catenin degradation [53]. UU-T02 is an inhibitor of β-catenin/TCF complex, although it has been proposed that structural modification of this compound may provide an even more potent nuclear inhibition of Wnt signaling [54]. As most of these compounds have not been shown as unequivocally effective to treat Wnt-related tumors, future studies using combination therapies that target multiple components of the pathway are likely to result in more potent and durable inhibition of Wnt-dependent cancers.

**Table 1:** List of synthetic and natural small molecules and their target protein on Wnt/β-catenin signaling pathway. * studies do not point out the specific target.

Protein	Flavonoids	Molecules	References
sFRP	ECGC	–	[55]
WIF-1	ECGC	–	[56,57,58,59]
Dsh	–	NSC668036; 3289-8625; FJ9; Pen-N3	[17,47,48,49]
CK1a		Pyrvinium	[39]
TNKS	Flavonone	XAV939; JW55; JW74; AZD2281; IWR-1; G007-LK; G244-LM	[40,41,42,43,44,60,61]
GSK-3β	Luteolin; Apigenin; Genistein	BIO	[62]
Destruction complex *	Kaempferol; Isorhamnetin; Baicalein	–	[63]
β-Catenin	Isoquercitrin	CCT031374; CCT036477; CCT070535	[53,64]
β-Catenin/TCF	Quercetin	iCRT3; iCRT5; iCRT14; PKF118–310; PKF115–584; CGP049090; BC21; UU-T02	[10,50,51,52,54,65]
CBP/β-catenin	–	ICG-001	[45]
p300/β-catenin	–	Windorphen	[46]
Unknown	Silibinin; Wogonin	–	[9,36]

## 4. Flavonoids and Wnt/β-Catenin Modulation in Colorectal Cancer

Flavonoids, polyphenolic compounds, constitute a very large group of natural products and one of the most characteristic classes of compounds in plants metabolism [66]. More than 6000 of these compounds are described and divided into these subclasses: flavones, chalcones, flavonols and flavanones [67]. The chemoprotective role of polyphenols against cancer has been extensively studied [68]. Evidences from case-control studies, cell culture and animal studies have shown a protective role against colorectal malignancy [68,69,70]. In the last years, studies have reported that anti-tumor effects promoted by flavonoids are related to the ability of these molecules to modulate the Wnt/β-catenin signaling pathway [6]. Moreover, the effects promoted by flavonoids have been detected in different parts of the signaling pathway, from the ligand receptor interaction (Wnt/Frizzled/LRP5/6) to the methylation of genes expressing pathway components, such as WIF (Wnt inhibitory factor 1) [4,65].

Quercetin is one of the most studied and has been submitted to clinical trials. Quercetin has been pointed as a potential anti-cancer drug in colorectal cancer [71,72] and its activity on colorectal cancer growth is closely related to Wnt modulation. Quercetin is able to interact with β-catenin and block binding between β-catenin and TCF [73,74]. Therefore, the treatment with quercetin inhibits Wnt/β-catenin in colorectal cancer cells *in vitro* [73,74]. More recently, it has been reported that quercetin, as well as the flavonoids luteolin and apigenin, inhibits GSK-3β [62]. This kinase is a constitutively acting multi-functional serine–threonine kinase involved in Wnt/β-catenin signaling pathway [20]. Our group also showed that quercetin is able to inhibit Wnt signaling pathway *in vivo* using *Xenopus* embryos as model system [65]. Many advances in the understanding of the mechanisms by which quercetin inhibits Wnt pathway have included this flavonoid in the list of bona fide Wnt/β-catenin inhibitors [75,76,77,78].

Another vastly studied anticancer flavonoid is EGCG, which inhibited Wnt signaling in colorectal cancer cells [74,79]. The mechanism by which EGCG acts on Wnt signaling is targeting another important protein that regulates this pathway. EGCG promotes *wif-1* gene (Wnt inhibitory factor 1) expression through its demethylation. WIF-1 is a Wnt antagonist that directly binds to Wnt molecules, blocking signaling [56,57,58,59]. Others studies also describe that treatment with EGCG reduced tumor multiplicity in the APC^−/+^ mouse, a model for intestinal tumorigenesis, by reducing nuclear β-catenin levels, suggesting an inhibition of the Wnt mediator β-catenin translocation to the nucleus [80,81]. Recently, ECGC also has been described to induce the expression of the Wnt inhibitor *SFRP1* gene in hepatoblastoma [55].

Ongoing studies of our group show that isoquercitrin (quercetin 3-*O*-β-d-glucopyranoside), a glycosylated derivative of quercetin, inhibits growth of different colorectal cancer cell lines (unpublished data). Genistein also suppressed β-catenin/TCF transcriptional activity in SW480 cells in a dose-dependent manner. This flavonoid affects upstream components of the β-catenin/TCF pathway by suppression of AKT phosphorylation, thus inhibiting GSK-3β dephosphorylation. Then, phosphorylated GSK-3β is able to phosphorylate β-catenin facilitating its ubiquitylation and degradation [63]. In 2010, Park and Choi showed that four flavonoids were able to inhibit Wnt/β-catenin signaling pathway. These authors demonstrated that kaempferol, isorhamnetin, genistein and baicalein inhibit Wnt in colorectal cancer through distinct mechanisms [63]. They suggested that kaempferol, baicalein and isorhamnetin affect Wnt upstream to β-catenin, and showed that genistein affects Wnt pathway by suppression of GSK-3β [63]. Interesting, the effect of flavonoids in colorectal cancer cells could be related to the type of mutation on Wnt/β-catenin. Silibinin treatment inhibited cell growth, induced cell death, and decreased levels of nuclear and cytoplasmic β-catenin in SW480 cell line, which harbors mutation in the *APC* gene. However, in HCT116 cells, which harbor wild-type APC but mutant β-catenin, silibinin has no effect, suggesting its selective effects on Wnt/β-catenin pathway [82]. Wogonin, a mono-flavonoid isolated from *Scutellaria radix*, a traditional Chinese medicine, has been shown to decrease expression of Wnt target genes *Wnt3A*, *LRP6*, *Cyclin D1*, *c-Myc* and to increase expression of Axin1 [83]. Wogonin also reduced TOPFLASH reporter activity on HCT116. Narwal M *et al.* showed flavonone as an inhibitor of Wnt signaling by modulating TNKS activity [60]. The authors tested different modifications on flavonone structure resulting in augmentation of its inhibitor potency [60], illustrating the importance of studying natural compounds, and using its natural structure as a model to novel and more potent compounds.

## 5. Conclusions

Despite of the significant advances in diagnostic of CRC, current treatments consist mostly of surgery and conventional chemotherapy. These treatments confer limited benefit, which makes CRC the third leading cause of cancer death among men and women. CRC development and progression involve silencing of genes and alteration of regulatory mechanisms in one or more members of the Wnt/β-catenin signaling pathway. Thus, the identification of substances capable of modulating the Wnt/β-catenin signaling has been a major effort for many research groups. Notably several new compounds have recently been described as inhibitors of different components of the Wnt/β-catenin signaling pathway. Particular attention has been given to flavonoids since case-control, cell culture and animal studies have revealed a plethora of bona fide compounds with protective role against CRC malignancy. However a long way has still to be paved to achieve treatment success of CRC. One problem to overcome is the bioavailability of flavonoids during cellular metabolism. In this regard, chemical structure modification and the use of nanoparticles may be a good strategy to develop more effective flavonoids to target Wnt/β-catenin and fight CRC growth and progression.

In summary, the recent advances in the understanding of the mechanisms of action of flavonoids targeting Wnt/β-catenin may shed light on future CRC therapies.
